# Supplementary material for: Syringin: A Phenylpropanoid Glycoside Compound in Cirsium brevicaule A. GRAY Root Modulates Adipogenesis
Source: Molecules. 2021 Mar 11;26(6):1531. doi: 10.3390/molecules26061531 (PMC7999402; doi:10.3390/molecules26061531)
Supplement: Supplementary file 1 [file molecules-26-01531-s001.pdf]

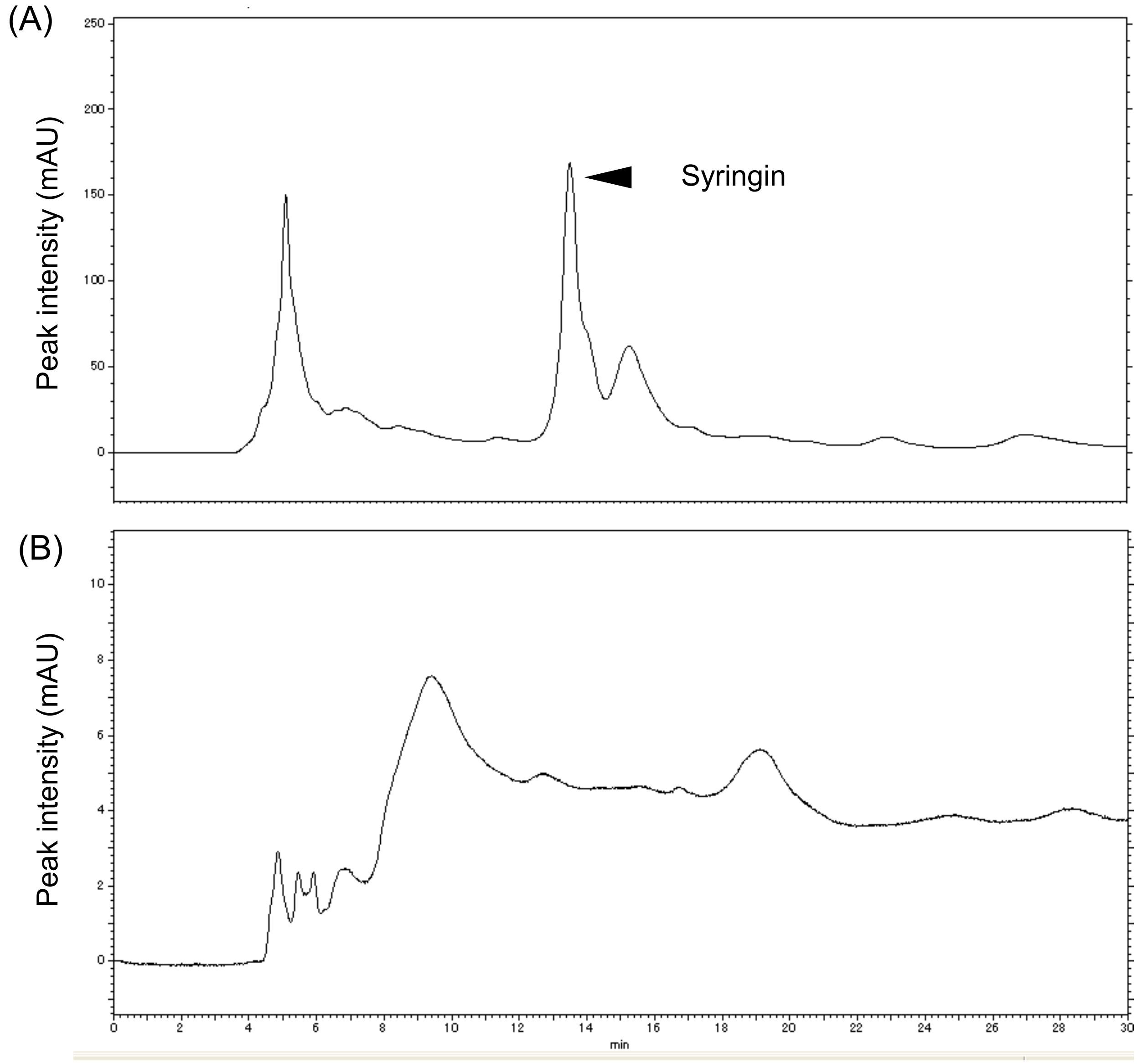

Supplementary Figure S1 HPLC chromatogram of CbR methanol extract fractions detected at 271 nm (**A**) Fr-1 and (**B**) Fr-2 (Develosil column 4.6 x 250 mm) Chloroform : Methanol 9:1

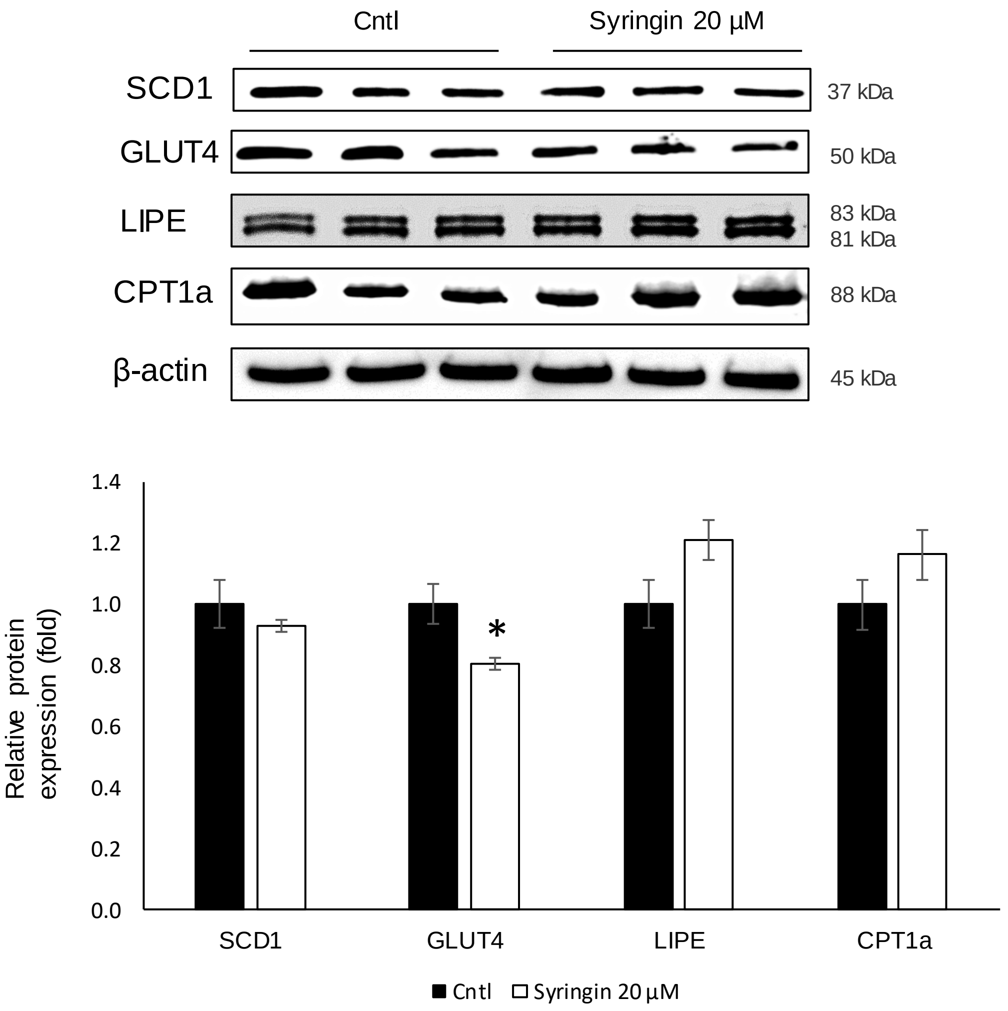

Supplementary Figure S2. Effect of syringin on the expression of lipogenesis and lipolysis-related proteins. The results are presented as means ± SEM of three independent experiments ($n = 3$). The asterisk (*) indicates a significant difference between control and treatment groups by Student's *t-test*. *$p < 0.05$ vs. control (Cntl).
